# Ubiquitination-related biomarkers in metastatic melanoma patients and their roles in tumor microenvironment

**DOI:** 10.3389/fonc.2023.1170190

**Published:** 2023-05-19

**Authors:** Li Zhang, Zhehao Shi, Fan Zhang, Bin Chen, Wei Qiu, Lei Cai, Xiaohua Lin

**Affiliations:** Department of Dermatology and Venereology, The First Affiliated Hospital of Wenzhou Medical University, Wenzhou, China

**Keywords:** skin cutaneous melanoma, immune, ubiquitination, prognostic signature, 4-URGs

## Abstract

**Background:**

Skin cutaneous melanoma (SKCM) is the deadliest type of cutaneous malignancy. Ubiquitination is a process of protein sorting and degradation that exhibits multiple functions in the progression of various tumors. This study aimed to characterize a set of genes for ubiquitination in SKCM.

**Methods:**

The expression patterns of ubiquitin-associated genes (URGs) and the corresponding clinical information in SKCM tissues were comprehensively analyzed based on The Cancer Genome Atlas (TCGA) database. We performed univariate and multivariate Cox proportional regression models to characterize the risk scores and identify four critical genes related to prognostic ubiquitination (HCLS1, CORO1A, NCF1 and CCRL2), which were used to construct the prognostic signatures. We also studied the effects of HCLS1, CORO1A and CCRL2 on tumor metastasis-related indicators at the cellular level through *in vitro* experiments.

**Results:**

SKCM patients in the low-risk group showing a longer survival than those in the high-risk group. Characteristic risk scores correlated with several clinicopathological variables and reflected the infiltration of multiple immune cells. In addition, the knockdown of CLS1, CORO1A and CCRL2 affected cellular malignant biological behavior through the EMT signaling pathway.

**Conclusion:**

This study provides a novel and prospective strategy to improve the clinical survival of SKCM patients.

## Introduction

Skin Cutaneous Melanoma (SKCM) is the most serious form of cutaneous tumor, and melanoma incidence is increasing in populations worldwide, especially in people over 60 years of age (1). SKCM is the most lethal of primary skin tumors. According to GLOBOCAN 2020, 324,635 new cases of melanoma were diagnosed worldwide in one year, accounting for 1.7% of all cancers, with 57,043 deaths and a melanoma-related mortality rate of 0.6% (2). As one of the most aggressive forms of skin cancer, melanoma has an aggressive metastatic growth pattern with metastasis occurring even in thin tumors and a poor prognosis (3). Currently, targeted therapy and immunotherapy are the mainstays of treatments for patients with metastatic melanoma (4). However, melanoma patients’ clinical progression and drug efficacy are very different, depending on the stage of diagnosis (5). How to better diagnose metastatic melanoma and understand the patterns of disease progression is still under investigation. It is critical to explore the tumor microenvironment of metastatic melanoma and to develop prognostic evaluation and therapy.

Post-translational modifications are procedures that alter the characteristics of proteins by adding or deleting modifying groups (6, 7). Ubiquitination is one of the most prevalent and important post-translational modifications in living organisms, and it is involved in homeostatic control as well as a variety of pathological processes (8–10). Ubiquitination has been linked to tumor formation and progression in a growing number of studies, and it is believed to play a critical role in cellular signaling pathways and biological activities in the microenvironment (11). For example, Nedd4 ubiquitinates VDAC2/3 to inhibit erastin-induced melanoma ferroptosis (12). FBXO32 links ubiquitination to epigenetic reprogramming of melanoma cells (13). TRIM15 and CYLD regulate ERK activation in melanoma through lysine-63-linked polyubiquitination (14). The prognostic prediction model of ubiquitination-related genes (URGs) in SKCM, on the other hand, has yet to be constructed. As a result, it is time to investigate the function of ubiquitination in metastatic melanoma.

In this work, we used bioinformatic analysis of melanoma data to examine the involvement of ubiquitination-associated genes in metastatic melanoma. According to our findings, ubiquitin-related genes (URGs) are potentially useful prognostic biomarkers and play a vital role in SKCM.

## Methods

### Data acquisition and data processing

The iUUCD 2.0 database contained 1366 URGs, in total. The TCGA database was mined for the 1326 ubiquitin-related genes’ accessible mRNA expression patterns. Based on the criterion of false discovery rate (FDR) 0.05 and |log2 Fold change (FC)| > 1, we also identified the differentially expressed URGs across primary and metastatic tumor samples. The R package “limma” was used to analyze differentially expressed URGs and the R package “pheatmap” was used to draw the heatmap (15). Consensus clustering analysis was performed for identifying ubiquitination-related molecular subtypes using R package “ConsensusClusterPlus” (16).

### Gene ontology analysis and functional enrichment analysis

GO analysis was used to evaluate the genetic properties of URGs, and the annotation of gene ontology categories comprised biological process (BP), cellular component (CC), and molecular function (MF). A gene set enrichment analysis was done to discover differences between patients in the high-risk and low-risk categories (GSEA). As reference gene sets, the GO and KEGG pathways gene sets were used (17). The “symbol” of differentially expressed URGs was converted into “entrezid” for enrichment analysis by R package “org.Hs.eg.db”. GO and KEGG pathway enrichment analysis were conducted by using the R package “clusterProfiler”. R packages “enrichplot” were used for visualization of the enrichment analysis results.

### PPI network construction

A tool for retrieving interacting genes (string; http://string-db.org) is available to describe and display proteomic correlations. Using the STRING database, we built a PPI network of potential URG genes to further evaluate meaningful URG candidates. Top genes by clustering analysis of differential genes with the Molecular Complex Detection (MCODE) plug-in.

### Correlation analysis

We employed the Pearson coefficient test to examine the relationship between 20 URG genes. The pearman coefficient test was also employed to examine the relationship between immune infiltrating cells. Lastly, we created correlation diagrams using the R packages “corrplot” to display the association of immune infiltrating cells, as well as 20 URG genes. Pearson correlation was used, and the correlation criteria was set at P 0.05.

### Univariate and multivariate cox analysis

To determine if URGs and risk models are prognostic variables for SKCM patients, we used univariate Cox regression to examine the connection between individual components and patient survival. We employed multifactorial COX to see if these parameters and models could be used to predict patient outcomes independently. At the same time, patients with SKCM were divided into low-risk and high-risk groups, and survival was calculated using Kaplan-Meier curves. The R package “Survival” was used to integrate the data of survival time. And the R package “MCPcounter” was performed to detect the abundance of 10 infiltrating immune cells between two clusters. Based on Monte-carlo sampling, inverse convolution p-values were calculated for all patients to provide reliability of the assessment.

### Cell culture

Short tandem repeat DNA profiling was used to confirm the authenticity of the B16 cell line, which was received from the Academy of Sciences Cell Bank of China. The Dulbecco’s modified Eagle’s medium (DMEM; Gibco, USA), which contains 10% heat-inactivated fetal bovine serum, was used to cultivate the cells.

### Real-time quantitative reverse transcription (qRT-PCR)

RNA was extracted using RNAeasy™ RNA Isolation Kit with Spin Column (R0027, Beyotime, CHN) and reverse with a PrimeScript RT Master Mix Perfect Real-Time kit (RR037A, Takara, CHN). PCR was performed using a standard SYBR Green PCR kit (RR820A, Takara, CHN). All reactions were performed in triplicate and the results were normalized to β-actin expression. The primers for dentification are 5’- AGTGGGCCATGATGTGTCTG -3’ (HCLS1 F), 5’-CTCCCCATCGTTGCTCCTTT-3’ (HCLS1 R), 5’-GCACCCAGACACGATCTACAG-3’ (CORO1A F), 5’-GGACGGTCCTTCTCAGCTAC-3’ (CORO1A R)5’-CCTGGTTGTGCTTATCCTGGT-3’ (CCRL2 F), 5’-AGAATTTTACACATGGGATCGCC-3’ (CCRL2 R), 5’-CCTGGCACCCAGCACAAT-3’ (β-ACTIN F), 5’- GGGCCGGACTCGTCATAC -3’ (18, 19).

### Cell transfection

The siRNAs were purchased from Shanghai Genepharma Company (Shanghai, China). The siRNAs were introduced into B16 cells with GP-transfect-Mate (Genepharma, Shanghai, China) for 48h, 72 h and empty vector was used as control. The target sequences of siRNA for HCLS1 were 5’- GCUGUCGGCUUCAAUGAAATT-3’(siHCLS1-1), 5’-GGCUGUAUAUGAUUACCAATT-3’(siHCLS1-2) and 5’- GAAGGAUAAAUGGGACAAATT-3’ (siHCLS1-3). The target sequences of siRNA for CORO1A were 5’-CAGGUUGUGACAACGUGAUTT-3’ (si CORO1A-1), 5’- CAGACACGAUCUACAGUGUTT-3’(si CORO1A-2) and 5’- CUCUCCAUGUUCAGUUCCATT-3’ (siCORO1A-3). The target sequences of siRNA for CCRL2 were 5’- GCGCCCUACAAUAUUGCAUTT-3’ (si CCRL2-1), 5’- GAUGGGACAUUUAGCAAAUTT-3’(si CCRL2-2) and 5’- CCCUGUGGCAUCAUUACAATT-3’ (si CCRL2-3) (20, 21).

### Cell viability assay

After transfection for 48 h, B16 cells were inoculated overnight into 96-well plates at a rate of 5 ×10^3^ cells per well. At 0, 24, 48, 72 and 96 h, 100 μL of culture medium and 10 μL of CCK-8 were added to each well, respectively. Optical density at 450 nm was measured by enzyme immunoassay.

### Cell colony formation assays

After transfection, 5 ×102 cells in culture medium were seeded into 12-well plates, and the medium was replaced every other 3-4 days. After 14 days, cells were stained with crystal violet. Following staining, the clone cells were imaged and counted.

### Cell migration and invasion assays

Cell migration analysis was performed using 24-well Transwell chambers (Corning, Corning, NY, USA). After 48h of transfection, 5×10^5^ B16 cells per well were resuspended in serum-free culture medium and grown into the upper chamber, and medium containing 10% fetal bovine serum was added to the lower chamber. After incubation for 48h, the cells were stained with crystal violet and photographed. For cell invasion assays, the operation was similar to that of migration assays except that the upper chamber was pre-coated with 1:9 Matrigel (BD Biosciences, Franklin Lakes, NJ, USA).

### Statistical analysis

The statistical analyses were conducted using GraphPad Prism 8.0 software (GraphPad Software, Inc.). Data were shown as mean ± standard deviation (SD). Comparison of continuous outcomes across multiple experimental groups was performed using a one-way analysis of variance (ANOVA) models. *P* value less than 0.05 was considered statistically significant.

## Results

### Differentially expressed URGs and metastasis-related ubiquitination genes

**Figure 1:**
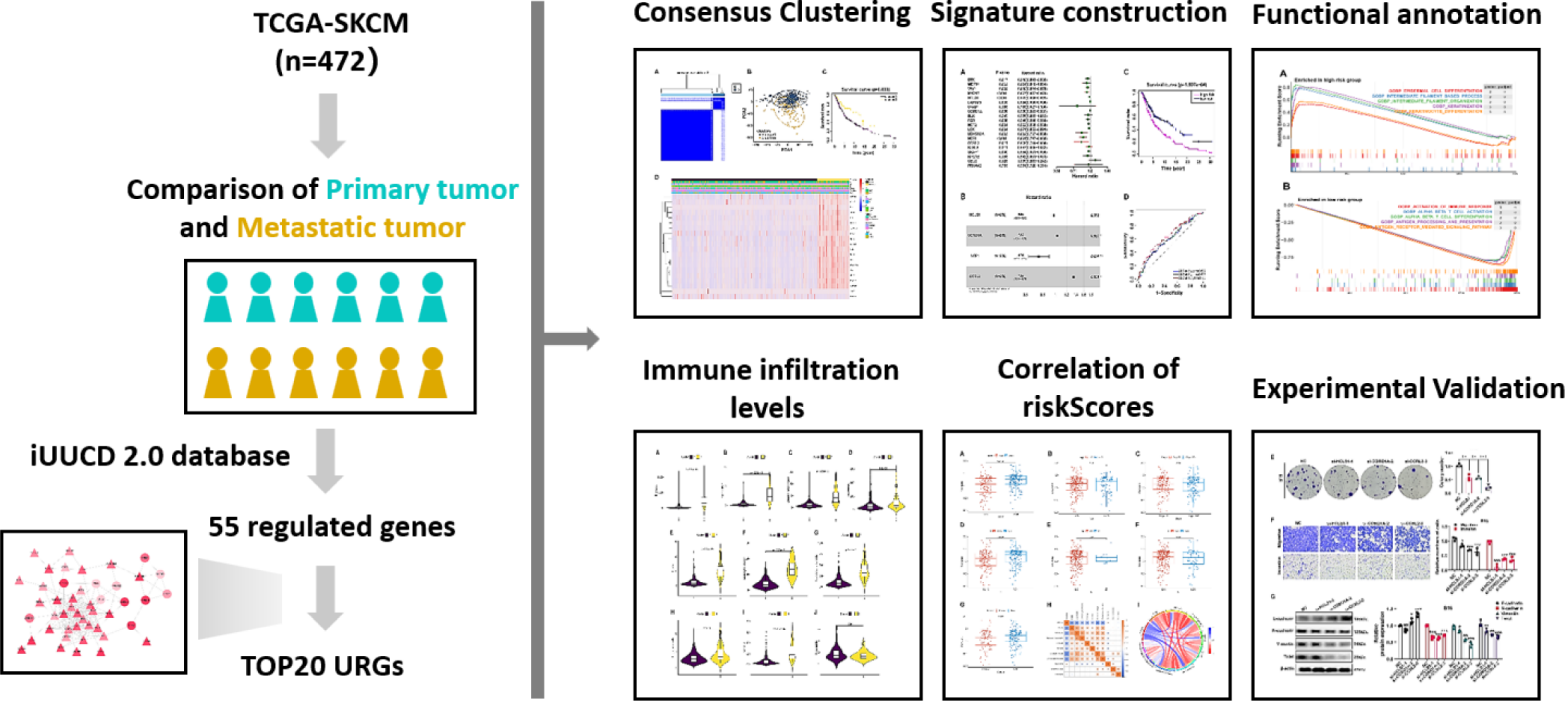
The workflow of the present study.

**Figure 2:**
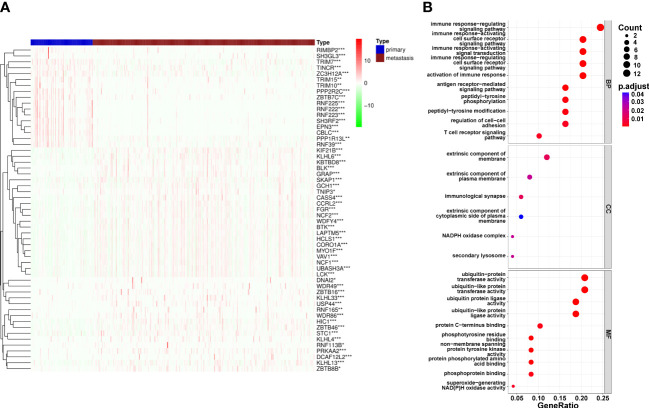
Differential expression of Ubiquitination‐related genes (URGs) between primary and metastatic tumor tissues. **(A)** clustering analysis of differentially expressed ARGs for TCGA database. **(B)** The general GO annotations for cellular component, molecular function, and biological processes; the horizontal axis represents the number of URGs under the GO term.

The flowchart was illustrated in our study (**Figure 1**). From the iUUCD 2.0 database, 1366 URGs in total were found (22). The TCGA database was mined for the 1326 ubiquitin-related genes’ accessible mRNA expression patterns. Additionally, we identified the differentially expressed URGs across primary and metastatic tumor samples using the criterion of |log2 Fold change (FC)| > 1 and false discovery rate (FDR) 0.05. By comparing 103 original tumors and 369 SKCM tissues, we were able to identify 55 differentially expressed URGs, including 17 down-regulated and 38 up-regulated genes, in order to ascertain if URGs may be employed as the markers to evaluate the prognosis of patients with metastatic melanoma (**Figure 2A**). We conducted functional enrichment analysis on these 55 genes to elucidate the probable biological roles of the aforementioned genes. The findings of GO analysis revealed that URGs genes were mostly linked to immunological pathways and regulation, as illustrated in **Figure 2B**. The highest enriched GO annotations were those that connected to the biological process’ immune response-regulating signaling route, immunological response-activating cell surface receptor signaling pathway, and immune response-activating signal transduction (BP). Extrinsic membrane components, extrinsic plasma membrane components, and immunological synapses are examples of cellular components (CC). The top three discovered molecular functions (MF) were ubiquitin-protein transferase activity, ubiquitin-like protein transferase activity, and ubiquitin-protein ligase activity.

### PPI network-based gene interaction analysis

**Figure 3:**
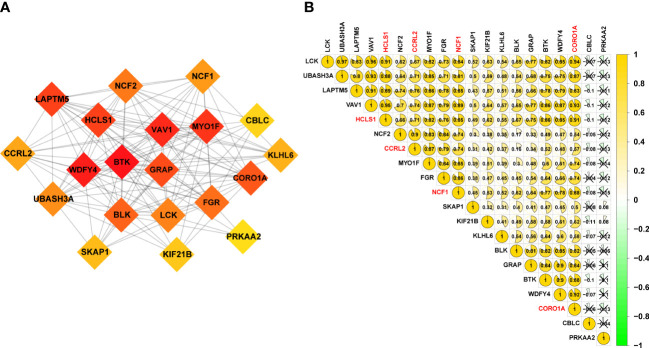
20 URGs correlation and interaction networks analysis. **(A)** The creation of the protein‐protein interaction (PPI) network for the 20 modulators of URGs. **(B)** Analysis of the correlation among 20 URGs. Green represents a negative correlation, and yellow represents a positive correlation.

To explore possible interactions between molecules associated to URGs, a PPI network was created. A subset of the top 20 genes was chosen to analyze the network using the MCODE method (**Figure 3A**). To look at how URGs in SKCM samples related to one another, we also ran Pearson correlation tests. The majority of ubiquitination-related genes had a strong positive correlation at 20 mRNA levels, as shown in **Figure 3B**. With the help of additional research, we want to validate our hypothesis that the downregulation of 20 URGs may contribute to the development of SKCM.

### Consensus clustering based on the levels of 20 genes’ expression

**Figure 4:**
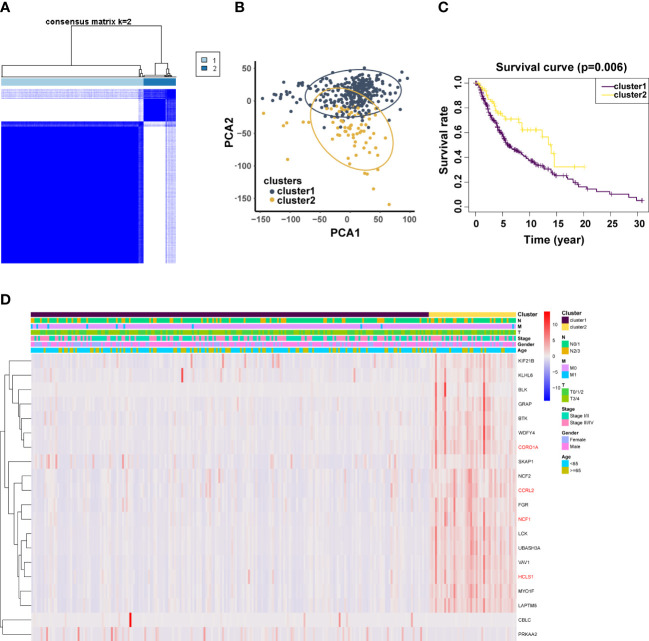
Validation of previously screened URGs genes. **(A)** Sample clustering heatmap of SKCm patients at k = 2 based on consensus clustering of 20 URGs. **(B)** Principal Coordinate Analysis (PCoA) scatter plot of SKCM patients based on 20 URGs in TCGA dataset. **(C)** T Associations between URGs‐based consistent clustering grouping (Cluster 1 and Cluster 2) and OS in SKCM patients. **(D)** Relationship between Clusters and clinical traits.

To investigate the relationship between crucial genes and metastasis and patient prognosis, 368 patients with metastatic melanoma were clustered by 20 related genes. Based on the level of group correlation, consensus clustering revealed that the samples could be classified into two categories (**Figure 4A**). Another classification approach (PCA analysis) was used for validation in order to validate the stability of this clustering in the categorization of SKCM patients in TCGA. The findings are displayed in **Figure 4B**. What’s more, we looked at patient survival in these two clusters and found a significant difference in survival probability between the two groups (p = 0.006). (**Figure 4C**). Additionally, we evaluated the clinical and pathological characteristics of these two groupings, including metastasis, tumor location, age, and gender, and discovered no discernible changes between them (**Figure 4D**).

### Identification of prognostic URGs and the OS related prognostic model

**Figure 5:**
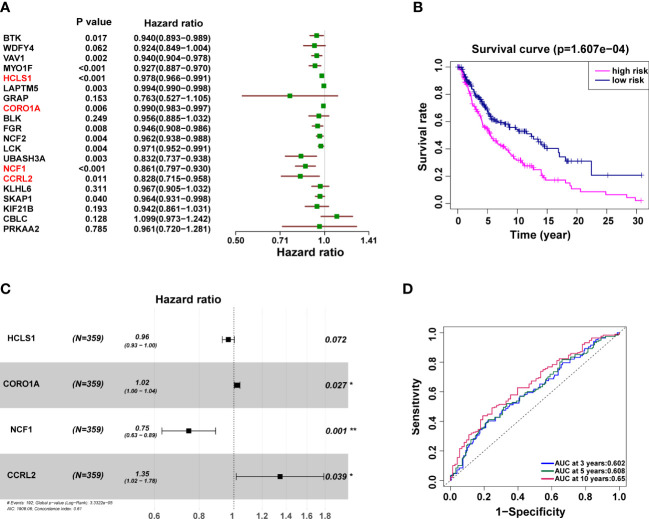
The independent prognostic significance of risk scores in SKCM was assessed using Cox regression. **(A, B)** Univariate **(A)** and multivariate **(B)** Cox regression analysis of overall survival for patients with SKCM. **(C)** Kaplan–Meier survival analysis curves between the low- and high-risk groups. **(D)** Training cohort’s ROC curve. The AUC for 3, 5, and 10 years was 0.602, 0.608 and 0.653, respectively.

To determine the connection between patient prognosis and various URGs expression patterns, we used univariate Cox regression analysis to analyze the TCGA data. Twenty URGs were concurrently substantially related to the outcome of SKCM patients (**Figure 5A**). A multivariate Cox regression analysis was then carried out (**Figure 5B**). OS risk genes included the four genes HCLS1 (HR 0.96, 95% CI 0.93-1.00; p=0.072), CORO1A (HR 1.02, 95% CI 1.00-1.04; p=0.027), NCF1 (HR 0.75, 95% CI 0.63-0.89; p=0.001), and CCRL2 (HR 1.35, 95% CI 1.02-1.78; p=0.039). It was decided to build the following prognostic model: risk score = (-0.03583 expression value of HCLS1) + (0.02082 expression value of CORO1A) + (-0.29007 expression value of NCF1) + (0.29699 expression value of CCRL2). The patients were split into subgroups of high-risk (n = 179) and low-risk (n = 180) patients according to the mean risk score from the training cohort. Patients were separated into high-risk (n = 179) and low-risk (n = 180) subgroups based on the mean risk score from the training cohort, as predicted, and the prognoses of these two groups were significantly different (**Figure 5C**). High-risk patients had a worse survival rate than low-risk individuals. The predictive performance of all SKCM patients in the training cohort was examined using time-dependent ROC curves based on the 4-URGs; the AUC for 3-, 5-, and 10-year OS was 0.602, 0.608, and 0.653, respectively (**Figure 5D**).

### Analysis of gene set enrichment between groups at high and low risk

**Figure 6:**
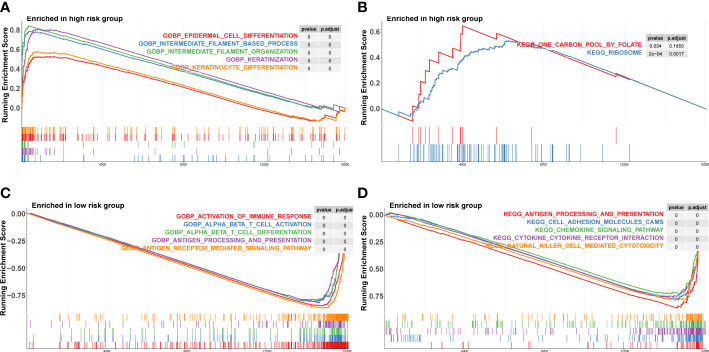
Gene set enrichment analysis in high‐risk and low‐risk groups. **(A, B)** KEGG pathways enriched in high‐risk group and low‐risk group. KEGG, Kyoto Encyclopedia of Genes and Genomes. **(C, D)** Biological process enriched in high‐risk group.

We conducted a GSEA analysis to investigate the biological differences across high-risk and low-risk groups. In the KEGG pathways, the key enriched pathways in the high-risk group were carbon pool by folate and ribosome (**Figure 6A**), whereas the main enriched pathways in the low-risk group were antigen processing and presentation, cell adhesion molecules, and chemokine signaling pathway (**Figure 6B**). In terms of biological processes, the high-risk group showed a higher concentration of keratinization, keratinocyte differentiation, intermediate filament-based process, intermediate filament organization, and epidermal cell differentiation (**Figure 6C**). The low-risk group exhibited enriched immune response activation, alpha-beta T cell activation, and antigen processing and presentation biological activities (**Figure 6D**).

### Tumor immune microenvironment analysis

**Figure 7:**
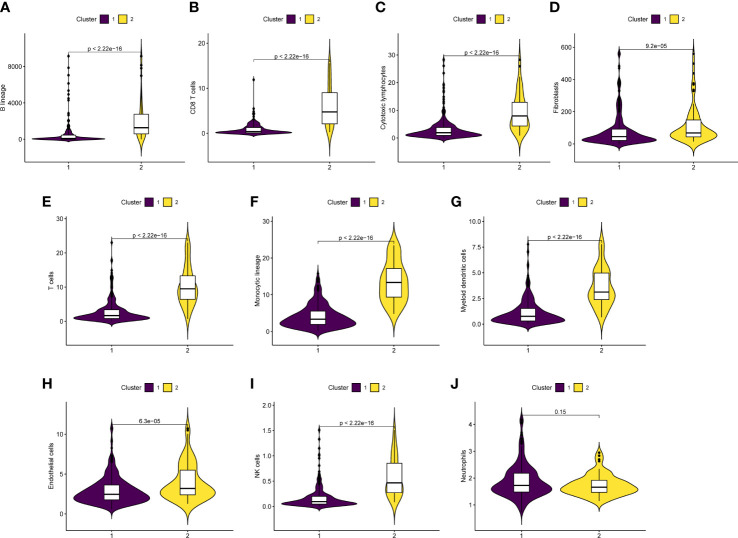
Correlation analysis of tumor immune microenvironment. **(A‐J)** Boxplot indicated that immune cell infiltration was higher in cluster 2 than in cluster 1.

Utilizing the “MCP counter” R package, immune cell infiltration in between two clusters was further proven in order to examine the functionality of URGs in the SKCM tumor microenvironment (**Figure 7**). B lineage, CD8 T cells, cytotoxic lymphocytes, fibroblasts, T cells, monocytic lineage, myeloid dendritic cells, endothelial cells, and NK cells were more prevalent in Cluster 2 than that in Cluster 1 (p<0.05) (**Figures 7A–I**). In the two clusters, neutrophils did not exhibit any obvious variations (p=0.15) (**Figure 7J**). According to the findings, cluster 2 had more immune cell infiltration.

### Role of risk correlation analysis in tumor microenvironment

**Figure 8:**
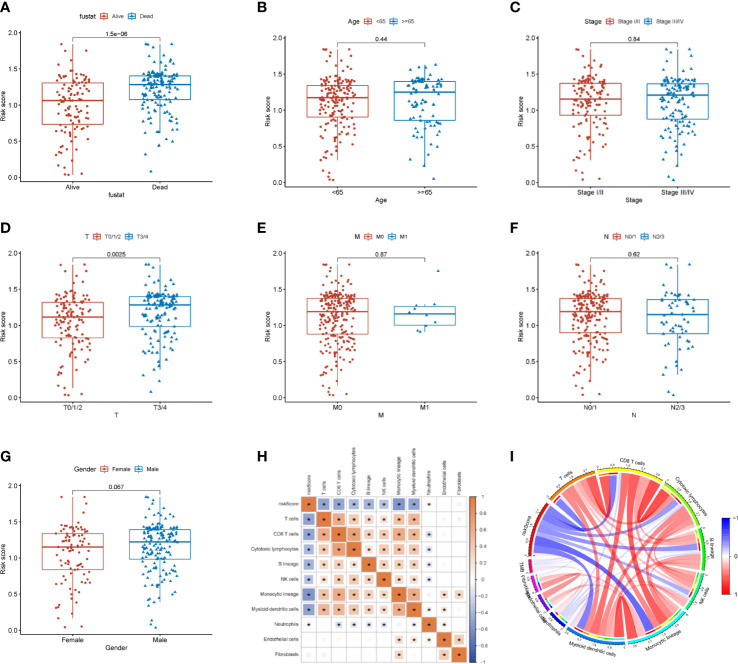
Relationship between the risk score and tumor immune microenvironment. **(A–G)** Correlation analysis between the risk score and pathological characteristics. **(H)** Correlation of the risk score with immune cells and immune function. **(I)** MCP counter algorithm analysis of the correlation between the risk score and immune cells. * p<0.05

In addition, we also examined the differences in pathological characteristics between risk scores. The results showed that patients with T3/4 (p=0.0025) and dead (p<0.05) had higher risk scores, but there was no significance in age (p=0.44), tumor stage (p=0.84), M0/1 (p=0.87), N1-4 (p=0.62) and gender (p=0.067) in patients with SKCM (**Figures 8A–G**). Further, we found that the risk scores were negatively correlated with the infiltration of T cells (R=- 0.492, p<0.001), CD8 T cells (R=-0.503, p<0.001), cytotoxic lymphocytes (R=-0.391, p<0.001), B lineage (R=-0.415, p<0.001), NK-cell (R=-0.316, p<0.001), monocytic lineage (R=-0.587, p<0.001), and myeloid dendritic cells (R=-0.494, p<0.001), but positively correlated with the infiltration of Neutrophils (R=0.105, p<0.05) (**Figures 8H, I** and **Figure S1**).

### HCLS1, CORO1A and CCRL2 mediated cellular viability, migration, and invasion in B16 cells

**Figure 9:**
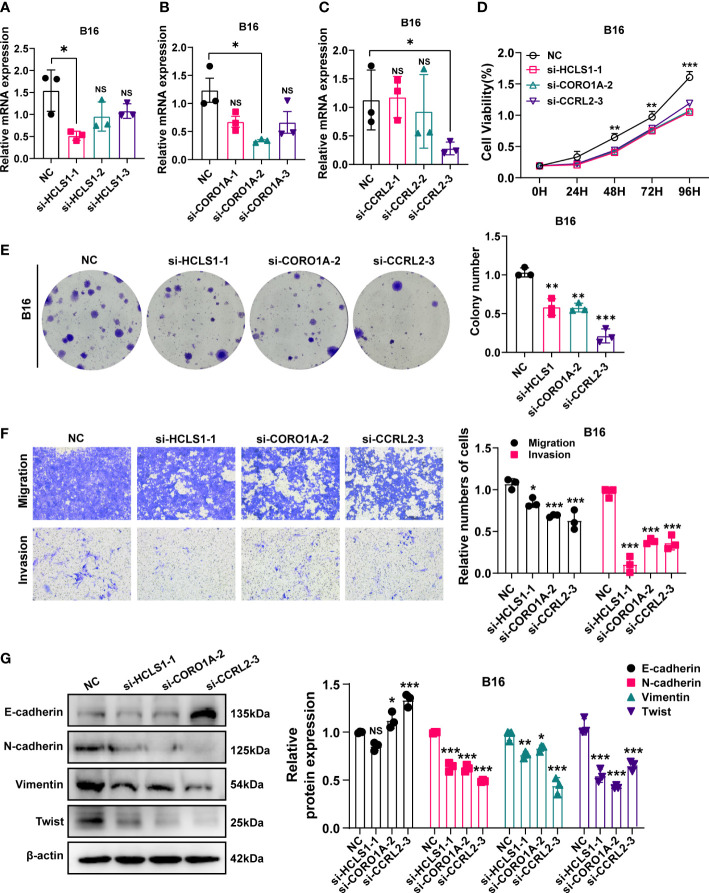
*In vitro* experiment after URGs knockdown. **(A–C)** PCR test. Then B16 cell Line was transfected with siRNA‐HCLS1, siRNA‐CORO1A, and siRNA‐ CCRL2. **(D)** CCK‐8 experiments. After URGs knockdown, the activity of B16 cell line decreased significantly. **(E)** After URGs knockdown, the cloning ability of B16 cell line decreased significantly. **(F)** Transwell assay. After URGs knockdown, the migration and invasion abilities of B16 cell line was significantly decreased. **(G)** Western‐blotting assay was performed to verify the knockdown efficiency of URGs and explore the relationship between URGs and EMT‐related proteins. Representative images and quantitative results were shown (n≥ 3). *P < 0.05, **P < 0.01, ***P <0.001.

NCF1 overexpression has been linked to melanoma lung metastases and colonization (23). As a result, through a series of *in vitro* tests, we further investigated the cellular molecular activities of HCLS1, CORO1A, and CCRL2. Firstly, qRT-PCR showed that in the B16 cell line, siRNA-HCLS1-1 significantly reduced HCLS1 expression (p=0.0139), siRNA-CORO1A-2 significantly reduced CORO1A expression (p = 0.0133), and CCRL2 was significantly reduced by siRNA-CCRL2-3 (p =0.0182) (**Figures 9A–C**). In the CCK-8 assay, HCLS1, CORO1A and CCRL2 knockdown significantly reduced the activity of B16 cells (p = 0.00049) (**Figure 9D**). In addition, the ability of B16 cell lines to form colonies was significantly reduced after HCLS1, CORO1A and CCRL2 knockdown, respectively (**Figure 9E**). Similarly, in Transwell assays, the migratory and invasive abilities of B16 cells were significantly reduced after HCLS1, CORO1A and CCRL2 knockdown (**Figure 9F**). Western blotting examined the downregulation of URGs in relation to EMT-related proteins. A statistically significant correlation was established between URGs and EMT proteins. When the HCLS1, CORO1A and CCRL2 genes were downregulated in the B16 cell line respectively, the expression of N-cadherin, Vimentin and Twist was significantly reduced, while the expression of E-cadherin was significantly upregulated (**Figure 9G**).

## Discussion

Melanoma is the most common cutaneous malignancy and difficult to treat, causing a significant toll on human health (24). Among them, metastatic melanoma is the most problematic, with an extremely low 5-year survival rate once diagnosed (25). The efficacy of existing conventional treatments for metastatic melanoma remains limited. The complicated immunological microenvironment and the highly metastatic character of melanoma are thought to be the primary causes of the poor prognosis and poor outcome (26). An intricate mechanism involving the interplay of several genes and signaling pathways in the tumor microenvironment underlies the continuing development and progression of SKCM (27). With this work, we aimed to pinpoint crucial genes involved in SKCM metastasis and further investigate the underlying processes.

A frequent post-translational protein change called ubiquitination has been linked to the development of cancer (28). Many essential enzymes involved in the ubiquitination pathway are currently thought to be attractive targets for cancer detection and therapy (29). Additionally, evidence for ubiquitination’s dual function in metastatic melanoma has been building over time (30). In order to ascertain their potential relevance in early diagnosis and prognosis determination in melanoma, athorough investigation of genes connected to the ubiquitination system is required.

This study investigates the role of URGs in metastatic melanoma. In the TCGA and iUUCD databases, we identified 55 URGs that were differentially expressed between primary and metastatic tumor samples. Subsequently, using the string database, correlations among the 55 URGs were analyzed and 20 TOP URGs were identified. Also, we applied consensus clustering based on the 20 URGs to classify SKCM patients with TCGA into two clusters. In addition, we investigated the relationship between the two clusters and the prognosis and the clinical characteristics of patients. We found that SKCM patients were predominantly classified as cluster 1, with cluster 1 having a lower prognosis than cluster 2. Furthermore, univariate and multifactorial Cox regression analyses were performed and four ubiquitin-related genes with prognostic significance were identified. And these four genes were used to create predictive features associated with ubiquitination in metastatic melanoma, which involved in metastasis regulation and influenced tumor progression in SKCM patients. Additionally, this feature may be used to calculate each patient’s risk score. A high-risk group and a low-risk group of SKCM patients in the cohort can be distinguished based on the median risk value, with the high-risk group having a much poorer prognosis than the low-risk group. For the prognosis and risk assessment of SKCM, this can be utilized as a guide. Immunotherapy is seen as a revolutionary advancement in the fight against cancer (31, 32).

Melanoma is an immunogenic tumor, and all of our current immunotherapies for melanoma have been clinically effective, but the efficacy in metastatic melanoma remains suboptimal (33, 34). Meanwhile, our understanding of the immune microenvironment of metastatic melanoma is not deep enough. There are limited data available regarding the immune microenvironment at different anatomical sites of melanoma metastases (35). Although immunotherapies showed the potential to improve the prognosis for metastatic melanoma patients, only a small group of patients can benefit from it (36). Multiple changes in the immune microenvironment have been identified as possible reasons for failure of immune checkpoint therapy (37). Therefore, more research on its immune microenvironment is needed to provide a basis for immunotherapy. Our study found that URGs were significantly enriched in immune-related pathways and that riskscore was negatively correlated with the infiltration of immune cells. Furthermore, immunological investigations have revealed that the quantity of immune infiltration varies between high-risk and low-risk individuals. It serves as a reference for immunological stratification of SKCM and aids in the direction of immunotherapy.

HCLS1 has been found to be associated with clinical prognosis and immune-correlation in osteosarcoma (38). CORO1A was associated with the TNM stage in non-small cell lung cancer (39). CORO1A was overexpressed in immune-rich tumors in ductal breast cancer and associated with clinical pathological factors (40, 41). One study revealed the critical role of CCRL2 in secondary acute myeloid leukemia (42). CCRL2 was reported to repress tumor growth *via* suppression of neoangiogenesis and chemerin concentration (43). CCRL2 expression as required for immune surveillance and anti-tumor immunotherapy in lung cancer (44). It has been reported that overexpression of CCRL2 was found in prostate cancer cells (45). Moreover, CCRL2 retarded invasion and chemotaxis through inhibition of p38 MAPK phosphorylation in breast cancer cells (46). Interestingly, CCRL2 overexpression enhanced invasion and migration of glioblastoma cells (47). Modulation of CCRL2 regulated proliferation, colony formation and migration of colorectal cancer cells (48). Dpletion of CCRL2 in mice enhanced melanoma tumor growth *via* impaired antitumor immunity and reduction of T cell responses (49). Ncf1, a single nucleotide polymorphic protein of neutrophil cytoplasmic factor 1, is essential for the formation of the NOX2 complex, a primary inducer of reactive oxygen species (ROS) (50). Tumor metastasis has been found to be closely related to Ncf1 gene polymorphisms that cause a poor ROS response. Mice deficient in NCF1 exhibit reduced melanoma growth and metastasis. Ncf1 promotes metastatic colonization of melanoma tumors *via* ROS (50). Ncf1 deficiency leads to accumulation of anti-tumor immune cells, which may mediate the molecular mechanism of reduced melanoma lung metastasis (23).

Based on the above, a number of studies have confirmed that ubiquitination associated high NCF1 expression in melanoma is closely associated with its metastasis, the role of HCLS1, CORO1A and CCRL2 in melanoma have not been fully elucidated, therefore we selected HCLS1, CORO1A and CCRL2 to investigate the malignant biological behavior in SKCM. In our study, HCLS1, CORO1A, CCRL2 and NCF1 are the genes in our constructs’ signatures and are associated with poor prognosis in SKCM. Our cellular assays showed that downregulation of URGs expression in B16 melanoma cells significantly reduced cell activity, proliferation, invasion and migratory capacity. EMT is a biological process that transforms epithelial cells into cells with a mesenchymal character. In clinical practice, poor prognosis of SKCM is frequently related with EMT. The expression of the epithelial marker E-cadherin was enhanced in the current study, whereas the expression of the mesenchymal indicators N-cadherin, Vimentin, and Twist was reduced, demonstrating knockdown of URGs potential to block the EMT process in B16 cells at the protein level. This adds to the evidence that URGs play a role in SKCM and that 4-URGs are potential targets for SKCM.

However, there are certain drawbacks to this study that must be addressed in the future. First, our work is based on a review of the TCGA database, and we analyzed the data retrospectively. A larger sample size is still required to confirm the results, and a prospective cohort, as well as the clinical samples, is required to further validate our model. Second, while URGs are unquestionably important in the development of SKCM, the processes behind them are unknown, and further information regarding the biological activities and molecular mechanisms of the four ubiquitination related genes in SKCM patients is needed. Furthermore, *in vivo* experiments are also very important. There is no *in vivo* experimental evidence that the identified genes play a role in metastasis, nor does it show a mechanistic link between the identified genes and EMT. The ex-vivo data are more relevant in the context of siRNA knockdown of *in vitro* regulated EMT markers and would be a stronger argument for the role of these genes in metastasis.

In conclusion, using a variety of bioinformatics techniques and experimental strategies, we successfully identified four key genes related to ubiquitination (HCLS1, CORO1A, CCRL2 and NCF1), thus providing new insights into the progress of SKCM.

## Data availability statement

The datasets presented in this study can be found in online repositories. The names of the repository/repositories and accession number(s) can be found in the article/**Supplementary Material**.

## Author contributions

Conceptualization, LZ. Software, ZS. Validation, ZS, LZ, and FZ. Resources, XL. Data curation, LZ, BC, and WQ. Writing—original draft preparation, ZS and LC. Writing—review and editing, XL. Supervision, LZ and XL. All authors contributed to the article and approved the submitted version.
