# Supplementary figures and images for: Ubiquitination-related biomarkers in metastatic melanoma patients and their roles in tumor microenvironment
Source: Front Oncol. 2023 May 19;13:1170190. doi: 10.3389/fonc.2023.1170190 (PMC10235493; doi:10.3389/fonc.2023.1170190)

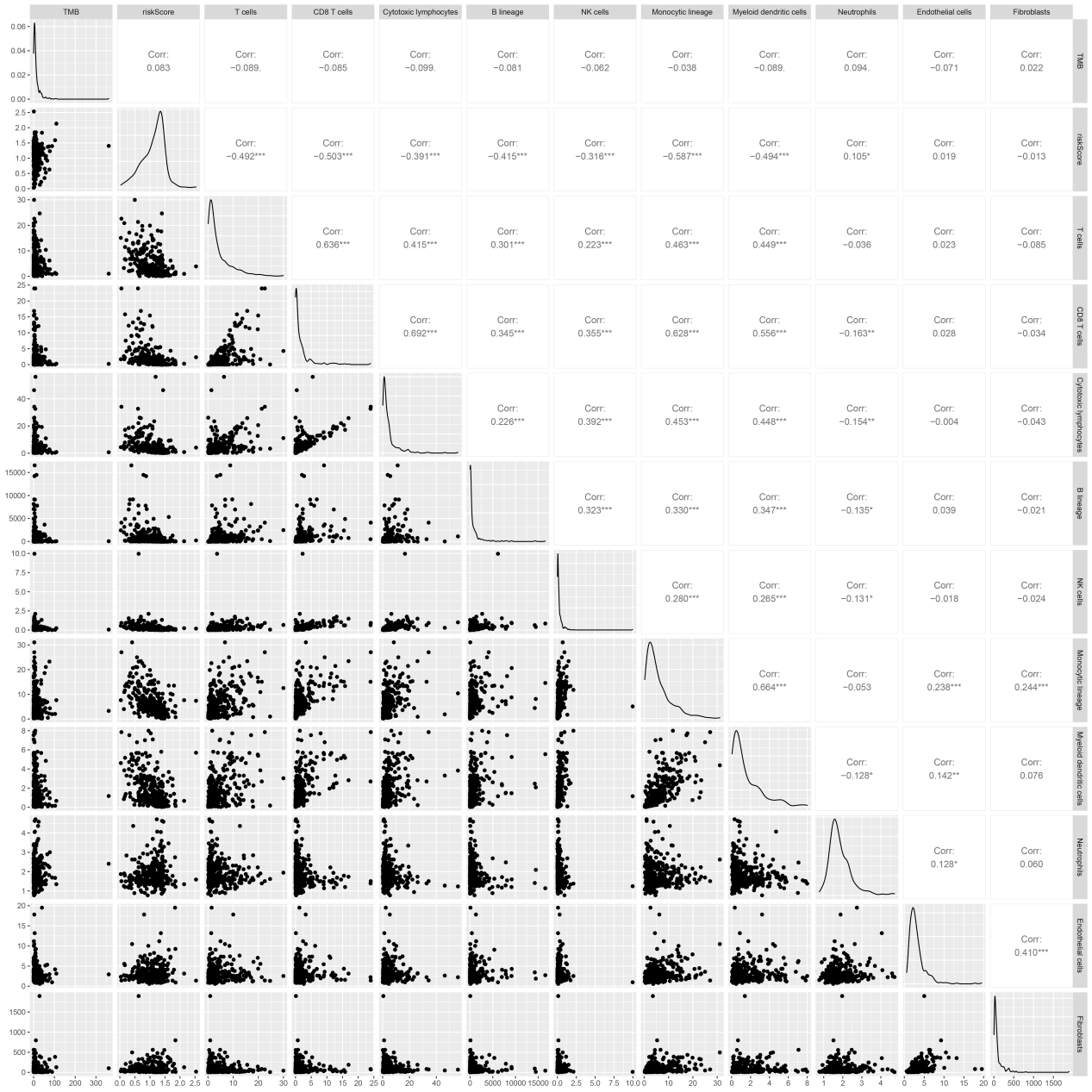


**Figure S1.** Relationship between risk score and immune cells.

Supplement: Supplementary file 1 [file DataSheet_1.docx]
